# Early Detection of Non-Small Cell Lung Cancer by Using a 12-microRNA Panel and a Nomogram for Assistant Diagnosis

**DOI:** 10.3389/fonc.2020.00855

**Published:** 2020-06-11

**Authors:** Weidong Wang, Dongni Chen, Weiwei Chen, Ziya Xin, Zirui Huang, Xuewen Zhang, Kexing Xi, Gongming Wang, Rusi Zhang, Dechang Zhao, Li Liu, Lanjun Zhang

**Affiliations:** ^1^Department of Thoracic Surgery, Sun Yat-sen University Cancer Center, Guangzhou, China; ^2^State Key Laboratory of Oncology in South China, Sun Yat-sen University Cancer Center, Collaborative Innovation Center for Cancer Medicine, Guangzhou, China; ^3^Department of Thoracic Surgery, School of Medicine, The First Affiliated Hospital, Zhejiang University, Hangzhou, China; ^4^Department of Anesthesiology, Sun Yat-sen University Cancer Center, Guangzhou, China; ^5^Department of Colorectal Surgery, Peking Union Medical College, National Cancer Center/Cancer Hospital, Chinese Academy of Medical Sciences, Beijing, China

**Keywords:** non-small cell lung cancer, miRNA, nomogram, liquid biopsy, early diagnosis

## Abstract

**Background:** We previously identified a 12-microRNA (miRNA) panel (miRNA-17, miRNA-146a, miRNA-200b, miRNA-182, miRNA-155, miRNA-221, miRNA-205, miRNA-126, miRNA-7, miRNA-21, miRNA-145, and miRNA-210) that aided in the early diagnosis of non-small cell lung cancer (NSCLC). We validated the diagnostic value of this miRNA panel and compared it with that of traditional tumor markers and radiological diagnosis. We constructed a nomogram based on the miRNA panel's results to predict the risk of NSCLC.

**Methods:** Eighty-two participants with pulmonary nodules on a CT scan and who underwent a pathological examination and surgical treatment were enrolled in our study. Patients were randomly divided into a training group or a validation group. The miRNA concentrations were quantified by RT-PCR and log-transformed for analysis. The cutoff value was determined in the training group and then applied in the validation group. A comparison between the miRNAs and traditional tumor markers [CEA, NSE, and cytokeratin 19 fragment 21-1 (Cyfra21-1)] and radiological diagnosis was performed. A nomogram based on the miRNA panel's results to predict the risk of NSCLC was constructed.

**Results:** The expression level of these 12 miRNAs was significantly higher in NSCLC patients than in benign patients. In the validation group, the specificity and positive predictive value were 96.4 and 95.8%, respectively, which were significantly higher than those using traditional tumor markers or radiological diagnosis. The sensitivity was 42.6%, which was also higher than that using tumor markers. Moreover, the sensitivity increased to 63.6% when the nodule diameters were larger than 2 cm. The miRNAs and seven clinical factors were integrated into the nomogram, and the calibration curves showed optimal agreement between the predicted and actual probabilities.

**Conclusions:** Our miRNA panel has clinical value for the early detection of NSCLC. A nomogram was constructed and internally validated, and the results indicate that it can assist clinicians in making treatment recommendations in the clinic.

## Introduction

Globally, lung cancer remains the leading cause of cancer incidence and mortality, with 2.1 million new lung cancer cases and 1.8 million deaths estimated in 2018, accounting for nearly one in five (18.4%) cancer-related deaths (1). Generally, lung cancers are classified into either small cell lung cancer (SCLC) or non-SCLC (NSCLC). NSCLC accounts for ~85% of all primary lung carcinomas, of which lung adenocarcinoma (LACA) is the most common histologic subtype (2).The 5-years relative survival for NSCLC was 23% for all stages combined, because nearly two-thirds of lung cancer (61%) are diagnosed at an advanced stage (3). Although the development of targeted therapy has greatly improved the prognosis of advanced lung cancer, only 10% of non-Asian and <40% Asian patients are appropriate for targeted therapy, and the rest of advanced-stage patients are still of poor survival outcome, which leads to only 4% for those with stage IV disease (4). Nevertheless, the 5-years survival rate for patients with small intrapulmonary cancers is 80% (5). Therefore, the identification of lung cancer at an early stage is essential for performing radical resection before the cancer is inoperable and improving survival.

In 2011, the National Lung Screening Trial (NLST) reported a 20% reduction in lung cancer mortality and a 6.7% decrease in all-cause mortality by screening patients with low-dose CT (LDCT) scans of the chest. However, a study reported that the overdiagnosis rate in the NLST was 18.5% (6). The relatively high false-positive rate of CT may cause some disadvantages, such as radiation exposure, high cost, and aggravated patient anxiety (7). Traditional tumor-associated antigens such as carcinoembryonic antigen (CEA), neuron-specific enolase (NSE), and cytokeratin 19 fragment 21-1 (Cyfra21-1) play poor roles in early detection of lung cancer because the sensitivity and specificity are not satisfied (8). Other invasive diagnostic methods such as needle biopsy or bronchoscope brush biopsy have a high incidence rate of complications and can take great discomfort to the patients (9). Therefore, it is essential to develop non-invasive and accurate biomarkers for NSCLC that could improve the accuracy of lung cancer screening and compensate for the deficiency in LDCT.

Liquid biopsy involves the analysis of cell-free nucleic acids and has the potential to make up the limitation of tissue-based biopsy especially for patients whose tissue analysis is inadequate on tissue specimens (10). After the first- or second-generation tyrosine kinase inhibitor (TKI) treatment, T790M mutation occurred in 50–65% of patients and finally led to drug resistance. For those patients, liquid biopsy is also a useful way in the detection of mutation with the benefits as non-invasiveness, easily accessible, and good repeatability (11). Some innovative approaches such as circulating tumor cells (CTCs), cell-free DNA (cfDNA), and tumor-associated autoantibodies are available for investigating lung cancer. Each of these approaches has its own advantages and shortcomings (10).

And in recent years, microRNAs (miRNAs), which are estimated to be 22 nucleotides in length, have been considered key components of small non-coding RNA transcripts. It has been reported that miRNAs are involved in the regulation of protein translation (12). Additionally, previous studies have demonstrated the irregular expression levels of miRNAs in several human malignancies. Alterations in miRNA expression patterns could play a role in the function of tumor suppressors and oncogenes (13). Thus, the plasma levels of miRNAs have been demonstrated as potential biomarkers for lung cancer risk and prognosis. However, most such studies had limited sample sizes or involved all pathological stages of lung cancer (14, 15). With the development of high-definition CT (HDCT), increasing numbers of small pulmonary nodules, especially pure ground-glass opacities (GGOs), have been detected, but these types of nodules are difficult to classify owing to limitations to CT imaging. Therefore, we first aimed to develop a panel of miRNAs that can be used as a non-invasive and highly accurate plasma marker for the early detection of lung cancer from small nodules. Then, we aimed to build a validated nomogram involving the miRNA panel and the clinical factors examined in our cohort to visualize the prediction and to aid in the precision of decision making.

## Methods

### Patients and Blood Samples

This study initially included 139 participants who had lung lesions on CT scan and underwent surgical treatment with pathological examination between July 2016 to March 2018 at Sun Yat-sen University Cancer Center. Blood samples were collected before operation from all 139 patients. Approval to use the plasma samples was obtained from an institutional review board, and a signed consent form was obtained from each patient. The study was approved by the Medical Ethics Committee and Institutional Review Board of Sun Yat-sen University Cancer Center, and the reference number is B2018-011.

**Figure 1:**
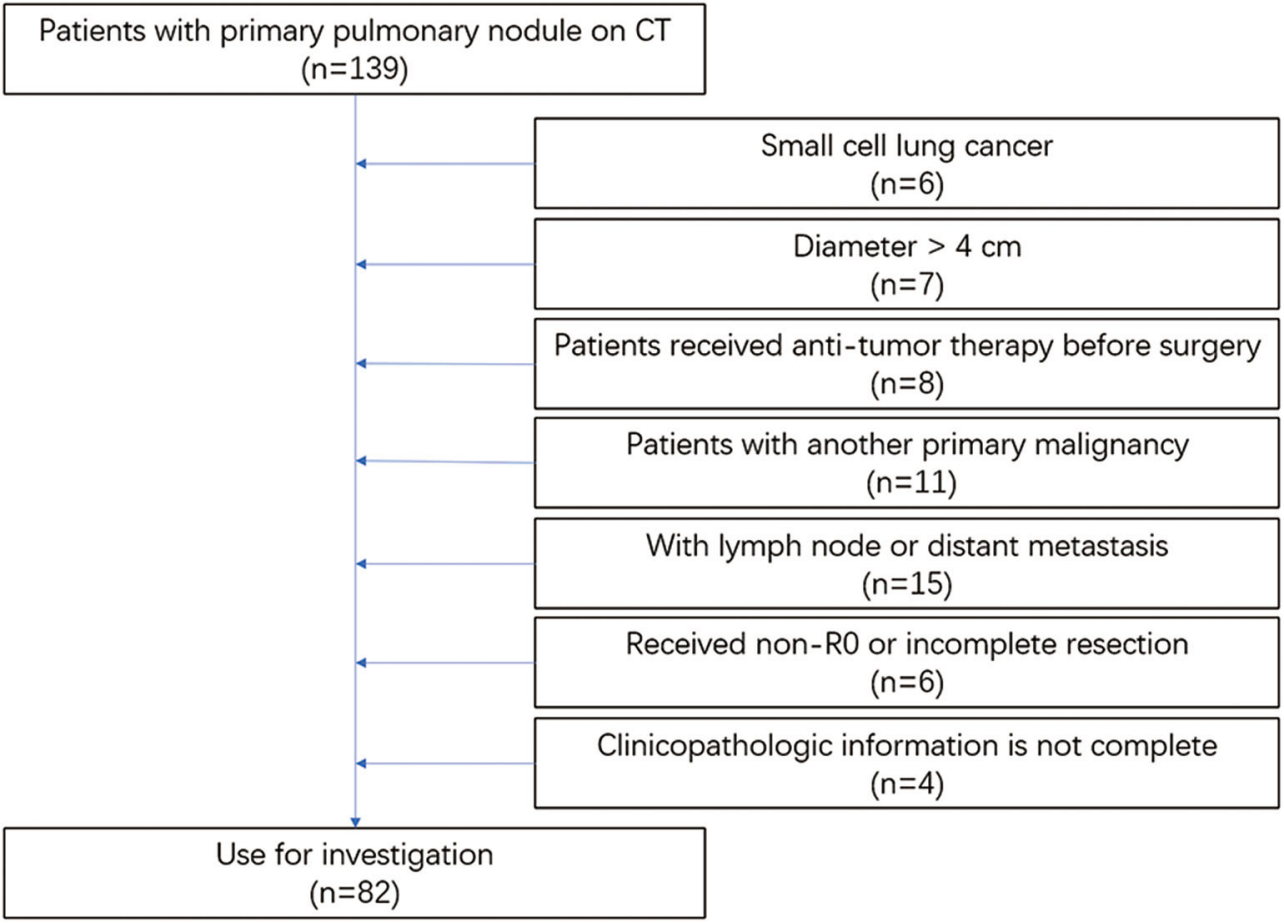
Flowchart of this study. CT, computed tomography.

The eligibility criteria were as follows: (1) patients with a pulmonary nodule on CT before the operation and postoperative pathology of NSCLC or benign disease; (2) preoperative examination showing no lymph node, regional, or distant metastasis; (3) lesion diameters of <4 cm on CT; (4) patients diagnosed with NSCLC could be accurately staged by a histopathological examination; and (5) patients had complete clinicopathological data. The exclusion criteria in this study were as follows: (1) patients with SCLC; (2) lesion diameters larger than 4 cm; (3) patients who received antitumor therapy before surgery; (4) patients with another primary malignancy; (5) patients with lymph node, regional, or distant metastasis; (6) patients who received non-R0 or incomplete resection; and (7) clinicopathological information was not complete (Figure 1).

Finally, 82 patients were further investigated. All participants were randomly assigned a random number, and those numbered 1–42 were allocated to the training group, whereas the remainder were allocated to the validation group.

### Quantitation of MicroRNAs in Blood Samples

Vacuum tubes containing RNA stabilization solution were adopted for the blood collection. Five milliliters of blood was collected from every participant before the operation, centrifuged within 2 h at 3,200 rpm for 10 min at 4°C, and then stored at −80°C before miRNA extraction.

Total RNA was extracted from 200 μl of blood samples using a miRNeasy Serum/Plasma Kit (Qiagen, USA) according to the manufacturer's instructions and eluted in a final volume of 14 μl. The total reaction volume for poly(A) tailing was 25 μl [eluted RNA, 10 μl; cel-miR-39, 1 × 10^9^ copies; 5 × PAP buffer solution, 4 μl; PolyA polymerase (Life, 74225Y/Z), 2–5 U; and the appropriate amount of RNase-free water to achieve a final volume of 25 μl]. The reaction conditions for poly(A) tailing were 37°C for 10–20 min and 65°C for 10 min. PolyA-tailed RNA (10 μl), reverse transcription (RT) buffer solution (2 μl), dNTPs (2 μl), reverse primers (20 μM), OmniScript (Qiagen Cat. No. 205111; 4 U), and RNase-free water (appropriate amount) were added to the RT reactions, and the final volume was 20 μl. The conditions for RT were as follows: cultivation at 37°C for 1 h, 85°C for 5 min, and holding at 4°C. The conditions for real-time polymerase chain reaction (PCR) were as follows: 95°C for 3 min, 40 cycles of 95°C for 15 s and 60°C for 35 s, and then 95°C for 15 s and 60°C for 60 s.

We used the OmniScript RT Kit (Qiagen, Germany) for the RT reaction. SYBR Green Mix (Qiagen, Cat. No. 208054) was used for real-time quantitative RT-PCR analysis to quantify the miRNAs. cel-miRNA-39 was chosen as an internal control.

### Statistical Analysis

The miRNA levels were log-transformed for analysis. The cutoff values for the miRNAs were defined as values greater than the mean plus 2 standard deviations (SDs) of the value of benign disease in the training group (16). These cutoff values were used to evaluate the diagnostic performance of the panel in the validation group.

The mean and SD were calculated to describe the quantification of the miRNAs. Categorical variables were calculated using the χ^2^ test and Fisher's exact test, whereas continuous variables were analyzed using the *t*-test, Mann–Whitney *U*-test, or Kruskal–Wallis test. The sensitivity and specificity were compared by matching χ^2^ tests. The area under the curve (AUC) and its standard error (SE) for the receiver operating characteristic (ROC) curve were used to evaluate the diagnostic value. Logistic regression was used to compare the respective AUCs and construct the nomogram. The performance of the nomogram was assessed by discrimination and calibration. For all analyses, two-sided *p* < 0.05 were considered significant. All analyses were performed using SPSS 20.0 software (IBM, Armonk, NY), GraphPad Prism 7.0 software (GraphPad software, La Jolla, CA), Med-calc 19.1 (Med-Calc software, Ostend, Belgium), and R 3.6.1 (The R Foundation for Statistical Computing, Vienna, Austria) with the rms statistical package.

## Results

### Study Population

A total of 82 patients were enrolled in our study and divided into a training group (*n* = 42) and a validation group (*n* = 40) by a table of random numbers. The clinical and pathological characteristics of our participants are shown in Table 1. A total of 28 patients with pathologically confirmed NSCLC, and 14 patients with benign tumors were included in the training group. The validation group comprised 26 patients with NSCLC and 14 patients with benign lung disease; 78.6% (33/42) patients in the training group and 65% (26/40) patients in the validation group had the smoking index <400, and 81% (34/42) patients in the training group and 75% (30/40) patients in the validation group had lesions with diameters ≤ 2 cm.

**Table 1:** Clinicopathological characteristics of the study population.

**Characteristic**	**Training group (*****n*** **=** **42)**			**Validation group (*****n*** **=** **40)**		
	**Lung cancer** **(*n* = 28)**	**Benign disease** **(*n* = 14)**	***p*-value**	**Lung cancer** **(*n* = 26)**	**Benign disease** **(*n* = 14)**	***p*-value**
**Age**, ***n*** **(%)**			*0.190*			*0.263*
>60 years	16 (38.1)	5 (11.9)		10 (25.0)	3 (7.5)	
≤ 60 years	12 (28.6)	9 (21.4)		16 (40.0)	11 (27.5)	
**Smoking Index**, ***n*** **(%)**			*1.000*			*0.945*
≥400	6 (14.3)	3 (7.4)		9 (22.5)	5 (12.5)	
<400	22 (52.4)	11 (26.2)		17 (42.5)	9 (22.5)	
**Sex**, ***n*** **(%)**			*0.029*			*0.481*
Male	10 (23.8)	10 (23.8)		16 (40.0)	7 (17.5)	
Female	18 (42.9)	4 (9.5)		10 (25.0)	7 (17.5)	
**Diameter**, ***n*** **(%)**			*0.137*			*0.433*
ϕ ≤ 1 cm	7 (16.7)	6 (14.3)		6 (15.0)	6 (15.0)	
1 < ϕ ≤ 2 cm	17 (40.5)	4 (9.5)		13 (32.5)	5 (12.5)	
ϕ > 2 cm	4 (9.5)	4 (9.5)		7 (17.5)	3 (7.5)	
**Composition**, ***n*** **(%)**			*0.005*			*0.120*
Pure GGO	11 (26.2)	6 (14.3)		10 (25.0)	3 (7.5)	
Mix GGO	10 (23.8)	0 (0.0)		8 (20.0)	2 (5.0)	
Solid nodule	7 (16.7)	8 (19.0)		8 (20.0)	9 (22.5)	
**Pathologic type**, ***n*** **(%)**			*NA*			*NA*
Adenocarcinoma	27 (96.4)	/		22 (84.6)	/	
SCC	0	/		3 (11.5)	/	
Other	1 (3.6)	/		1 (3.8)	/	
**Differentiation**			*NA*			*NA*
High	6 (22.2)	/		3 (14.3)	/	
Middle	16 (59.3)	/		17 (81.0)	/	
Low	5 (18.5)	/		1 (4.8)	/	
**Stage**, ***n*** **(%)**			*NA*			*NA*
IA1	4 (14.3)	/		6 (23.1)	/	
IA2	15 (53.6)	/		11 (42.3)	/	
IA3	2 (7.1)	/		4 (15.4)	/	
IB	7 (25.0)	/		5 (19.2)	/	

*GGO, ground-glass opacity; NA, not available; SCC, squamous cell carcinoma*.

### Reactivity of the MicroRNA Panel in the Training Set

**Figure 2:**
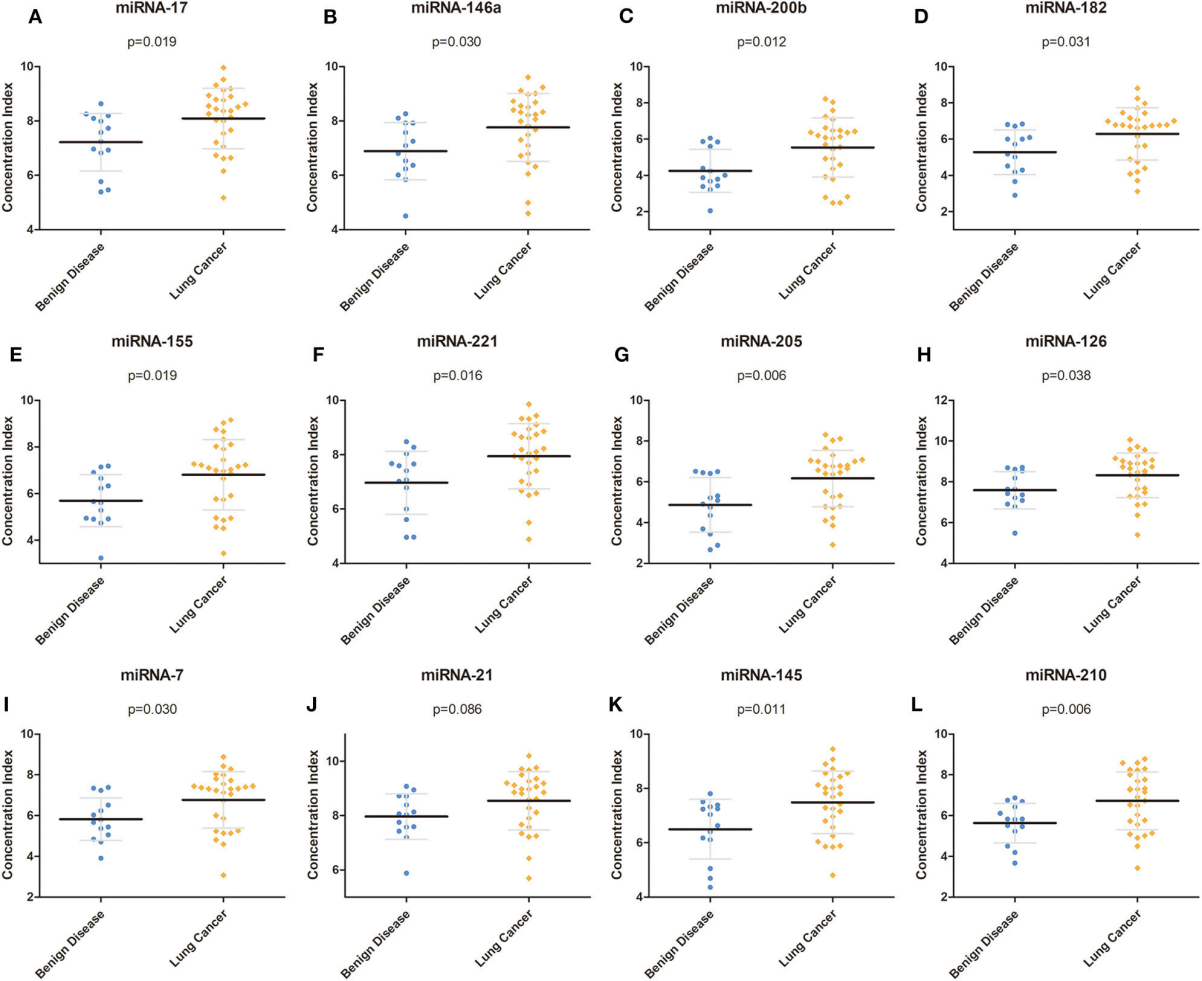
Concentration of each miRNA in the training group. **(A–L)** Concentrations of miRNA-17, miRNA-146a, miRNA-200b, miRNA-182, miRNA-155, miRNA-221, miRNA-205, miRNA-126, miRNA-7, miRNA-21, miRNA-145, and miRNA-210 in the training group. miRNA, microRNA.

To determine the reactivity of the miRNA panel, 12 miRNAs (miRNA-17, miRNA-146a, miRNA-200b, miRNA-182, miRNA-155, miRNA-221, miRNA-205, miRNA-126, miRNA-7, miRNA-21, miRNA-145, and miRNA-210) were identified from more than 100 cancer-related miRNAs, and the average expression level of these 12 miRNAs was quantified using RT-PCR in independent plasma samples. The results demonstrated that the miRNA expression level of miRNA-17, miRNA-146a, miRNA-200b, miRNA-182, miRNA-155, miRNA-221, miRNA-205, miRNA-126, miRNA-7, miRNA-145, and miRNA-210 was significantly higher in patients with NSCLC than in patients with benign disease (*p* = 0.019, *p* = 0.030, *p* = 0.012, *p* = 0.031, *p* = 0.019, *p* = 0.016, *p* = 0.006, *p* = 0.038, *p* = 0.030, *p* = 0.011, and *p* = 0.006, respectively), whereas the expression level of miRNA-21 was similar between the two groups (*p* = 0.086). The detailed results are shown in Table 2 and Figure 2. All 12 miRNAs showed significant discriminative ability between NSCLC and benign disease on the basis of the ROC curves (Figure S1). Among these 12 miRNAs, miRNA-205 had the largest AUC of 0.770 whereas miRNA-21 had the smallest AUC of 0.698.

**Table 2:** Concentration of each miRNA in the training and validation groups.

**MiRNAs** **(mean ±*SD*)**	**Training group (*****n*** **=** **42)**			**Validation group (*****n*** **=** **40)**		
	**Lung cancer** **(*n* = 28)**	**Benign disease** **(*n* = 14)**	***p*-value**	**Lung cancer** **(*n* = 26)**	**Benign disease** **(*n* = 14)**	***p*-value**
MiRNA-17	8.09 ± 1.11	7.22 ± 1.06	*0.019*	8.44 ± 0.85	7.71 ± 1.06	*0.022*
MiRNA-146a	7.76 ± 1.25	6.89 ± 1.05	*0.030*	8.05 ± 0.88	7.25 ± 1.14	*0.018*
MiRNA-200b	5.54 ± 1.63	4.24 ± 1.19	*0.012*	5.91 ± 1.54	4.58 ± 1.84	*0.020*
MiRNA-182	6.29 ± 1.44	5.28 ± 1.23	*0.031*	6.69 ± 1.29	5.75 ± 1.38	*0.039*
MiRNA-155	6.80 ± 1.51	5.69 ± 1.12	*0.019*	7.27 ± 1.50	6.12 ± 1.53	*0.027*
MiRNA-221	7.94 ± 1.20	6.97 ± 1.16	*0.016*	8.17 ± 0.87	7.42 ± 1.08	*0.023*
MiRNA-205	6.17 ± 1.39	4.87 ± 1.33	*0.006*	6.56 ± 1.29	5.61 ± 1.42	*0.039*
MiRNA-126	8.32 ± 1.10	7.59 ± 0.91	*0.038*	8.51 ± 0.85	7.87 ± 1.01	*0.039*
MiRNA-7	6.77 ± 1.38	5.83 ± 1.05	*0.030*	7.09 ± 1.12	6.10 ± 1.31	*0.017*
MiRNA-21	8.54 ± 1.07	7.97 ± 0.84	*0.086*	8.55 ± 1.24	8.12 ± 0.98	*0.273*
MiRNA-145	7.49 ± 1.15	6.50 ± 1.10	*0.011*	7.73 ± 0.95	7.05 ± 1.13	*0.049*
MiRNA-210	6.73 ± 1.42	5.63 ± 0.97	*0.006*	7.12 ± 1.18	6.11 ± 1.27	*0.016*

*SD, standard deviation; miRNA, microRNA*.

### Evaluation of the Reactivity and Cutoff Value of the MicroRNA Panel in the Validation Group

To further evaluate the expression of our miRNA panel, we also measured the expression level of miRNAs in the validation group. Similar to the training group, with the exception of miRNA-21, which was not significantly different between lung cancer and benign disease, the 11 remaining miRNAs were significantly higher in patients with lung cancer than in patients with benign disease (Table 2).

For convenience in the clinic, we transformed the expression level of the miRNAs to dichotomous data. Based on the expression levels of the 12 miRNAs in the training group, we formulated a cutoff value to distinguish lung cancer from benign disease. According to this cutoff value and the expression level of the miRNAs, patients in the validation group were further divided into a “miRNA positive group” and a “miRNA negative group.” Our results demonstrated that under the cutoff value formulated with the training group, the expression level of our miRNA panel was significantly associated with pathological type and differentiation (Table 3).

**Table 3:** Correlation between miRNA expression levels and clinicopathological characteristics.

**Characteristic**	**MiRNA panel expression level**		
	**MiRNA positive**	**MiRNA negative**	***p*-value**
**Age**, ***n*** **(%)**			*0.706*
>60 years	4 (10.0)	9 (22.5)	
≤ 60 years	10 (25.0)	17 (42.5)	
**Sex**, ***n*** **(%)**			*0.974*
Male	8 (20.0)	15 (37.5)	
Female	6 (15.0)	11 (27.5)	
**Diameter**, ***n*** **(%)**			*0.881*
ϕ ≤ 1 cm	5 (12.5)	7 (17.5)	
1 < ϕ ≤ 2 cm	5 (12.5)	13 (32.5)	
ϕ > 2 cm	4 (10.0)	6 (15.0)	
**Composition**, ***n*** **(%)**			*0.099*
Pure GGO	7 (17.5)	6 (15.0)	
Mix GGO	3 (7.5)	7 (17.5)	
Solid nodule	4 (10.0)	13 (32.5)	
**Pathological type**, ***n*** **(%)**			*0.006*
Malignant	13 (32.5)	13 (32.5)	
Benign	1 (2.5)	13 (32.5)	
**Differentiation**, ***n*** **(%)**			*0.037*
High	3 (14.3)	0	
Middle	7 (33.3)	10 (47.6)	
Low	0	1 (4.8)	
**Stage**, ***n*** **(%)**			*0.862*
IA1	3 (11.5)	3 (11.5)	
IA2	6 (23.2)	5 (19.3)	
IA3	1 (3.8)	3 (11.5)	
IB	3 (11.5)	2 (7.7)	

*CM, centimeter; GGO, ground-glass opacity; miRNA, microRNA*.

### Diagnostic Value of the MicroRNAs in the Validation Group

**Figure 3:**
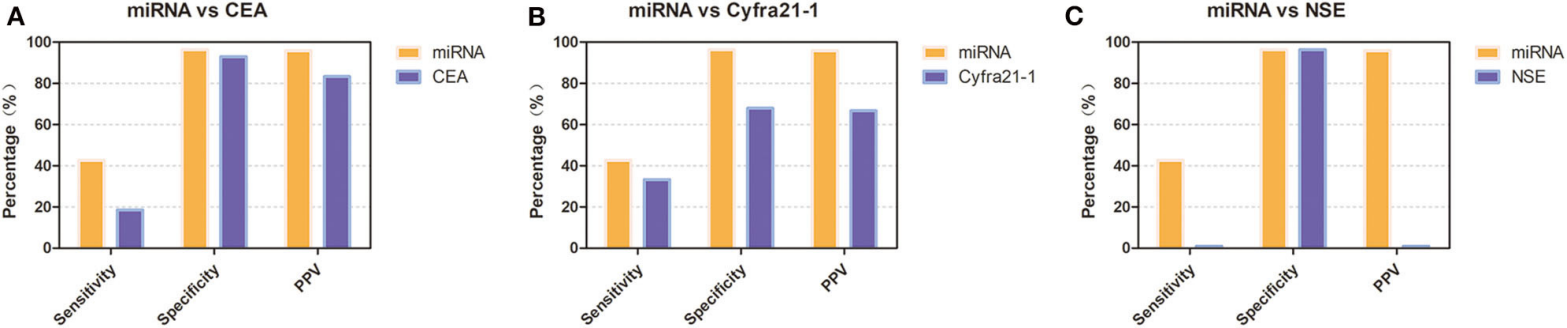
Diagnostic performance of the 12-miRNA panel compared with that of traditional tumor markers in the validation group. **(A)** Diagnostic performance of the 12-miRNA panel compared with that of CEA. **(B)** Diagnostic performance of the 12-miRNA panel compared with that of Cyfra21-1. **(C)**. Diagnostic performance of the 12-miRNA panel compared with that of NSE. *CEA*, carcinoembryonic antigen; *NSE*, neuron-specific enolase; *PPV*, positive predictive value; miRNA, microRNA; Cyfra21-1, cytokeratin 19 fragment 21-1.

To verify the diagnostic performance of these 12 miRNAs in distinguishing NSCLC from benign disease, we evaluated the sensitivity, specificity, and positive predictive value (PPV) of all 12 miRNAs according to the cutoff value formulated with the training group. The sensitivity of a single miRNA ranged from 11.5 to 38.5% in NSCLC patients. The specificity was 92.9% for miRNA-200b, miRNA-221, miRNA-126, and miRNA-210 in benign disease and 100.0% for the remainder of the miRNAs. The PPV of each single miRNA ranged from 75.0 to 100.0%. Concerning dichotomous data, the AUC of each single miRNA ranged from 0.541 to 0.692 (Table 4). The combined AUC for all 12 miRNAs was 0.714 (*p* < 0.001), with a sensitivity of 50.0% and a specificity of 92.9% (Figure S2), which were higher than those when using each single miRNA alone. Interestingly, we compared the diagnostic value of our miRNA panel with that of traditional tumor markers (CEA, Cyfra21-1, and NSE). The miRNA panel showed significantly higher sensitivity than CEA and NSE, a higher PPV than Cyfra21-1 and NSE, and higher specificity than Cyfra21-1 (Figure 3 and Table S1).

**Table 4:** Sensitivity, specificity, positive predictive value, and area under the curve of the 12 miRNAs in the validation group.

**MiRNAs**	**Sensitivity (%)**	**Specificity (%)**	**PPV (%)**	**AUC (95% CI)**	***p*-value**
MiRNA-17	11.5	100.0	100.0	0.558 (0.375–0.741)	*0.552*
MiRNA-146a	11.5	100.0	100.0	0.558 (0.375–0.741)	*0.552*
MiRNA-200b	34.6	92.9	90.0	0.637 (0.464–0.810)	*0.156*
MiRNA-182	19.2	100.0	100.0	0.596 (0.419–0.773)	*0.321*
MiRNA-155	38.5	100.0	100.0	0.692 (0.532–0.853)	*0.047*
MiRNA-221	11.5	92.9	75.0	0.522 (0.334–0.710)	*0.821*
MiRNA-205	19.2	100.0	100.0	0.596 (0.419–0.773)	*0.321*
MiRNA-126	15.4	92.9	80.0	0.541 (0.356–0.727)	*0.671*
MiRNA-7	19.2	100.0	100.0	0.596 (0.419–0.773)	*0.321*
MiRNA-21	15.4	100.0	100.0	0.577 (0.397–0.757)	*0.427*
MiRNA-145	11.5	100.0	100.0	0.558 (0.375–0.741)	*0.552*
MiRNA-210	34.6	92.9	90.0	0.637 (0.464–0.810)	*0.156*
12-miRNA panel	50.0	92.9	92.9	0.714 (0.540–0.852)	* <0.001*

*AUC, area under the curve; CI, confidence interval; PPV, positive predictive value; miRNA, microRNA*.

**Figure 4:**
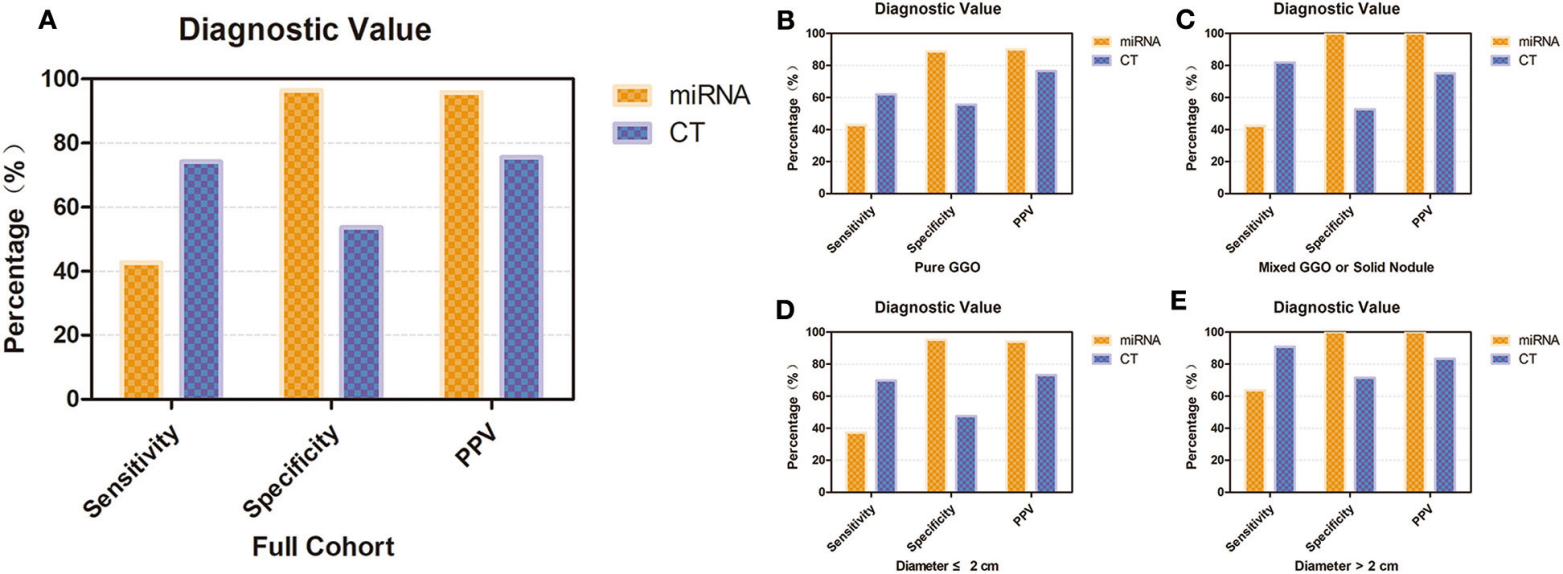
Diagnostic value of the 12-miRNA panel compared with that of CT diagnosis. **(A)** Diagnostic value of the 12-miRNA panel compared with that of CT diagnosis in the whole validation group. **(B)** Diagnostic value of patients who have a pure GGO. **(C)** Diagnostic value of patients who have a mixed GGO or solid nodule. **(D)** Diagnostic value of patients whose nodule diameter was ≤ 2 cm. **(E)** Diagnostic value of patients whose nodule diameter was larger than 2 cm. CT, computed tomography; GGO, ground-glass opacity; PPV, positive predictive value; miRNA, microRNA.

We also conducted subgroup analyses to investigate the diagnostic value of our miRNA panel compared with that of CT diagnosis in patients with different lesion diameters and solid proportions. In the whole validation group, our results revealed that although the miRNA panel was not as sensitive as CT diagnosis (42.6 vs. 74.1%, *p* = 0.020), the miRNA panel had significantly higher specificity and a significantly higher PPV than had CT diagnosis (96.4 vs. 53.6%, *p* < 0.001; 95.8 vs. 75.5%, *p* < 0.001, respectively) (Figure 4A). It is interesting that the sensitivity of CT diagnosis decreased sharply from 81.8 to 61.9% with the reduction in the solid proportion, but it remained stable for the miRNA panel with different solid proportions. The sensitivity of the miRNA panel increased from 37.2 to 63.6% when the lesion diameters were larger than 2 cm. The detailed results are summarized in Figure 4 and Table S2.

### Predictive Nomogram for Non-Small Cell Lung Cancer Probability Based on the MicroRNA Panel and Other Preoperative Clinical Characteristics

**Figure 5:**
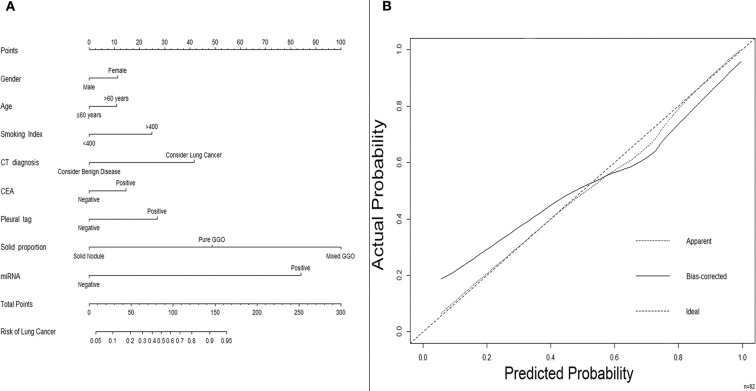
A nomogram predicting the risk of non-small cell lung cancer. **(A)** A nomogram predicting the probability of non-small cell lung cancer. The value of each factor is given a score on the point scale axis. The total score can be calculated by adding every single score and projecting the total score to the total point scale so that clinicians can estimate the probability of NSCLC. **(B)** Calibration curves for the nomogram. The *x*-axis represents the probability predicted by the nomogram, and the *y*-axis represents the actual probability of NSCLC. NSCLC, non-small cell lung cancer.

**Figure 6:**
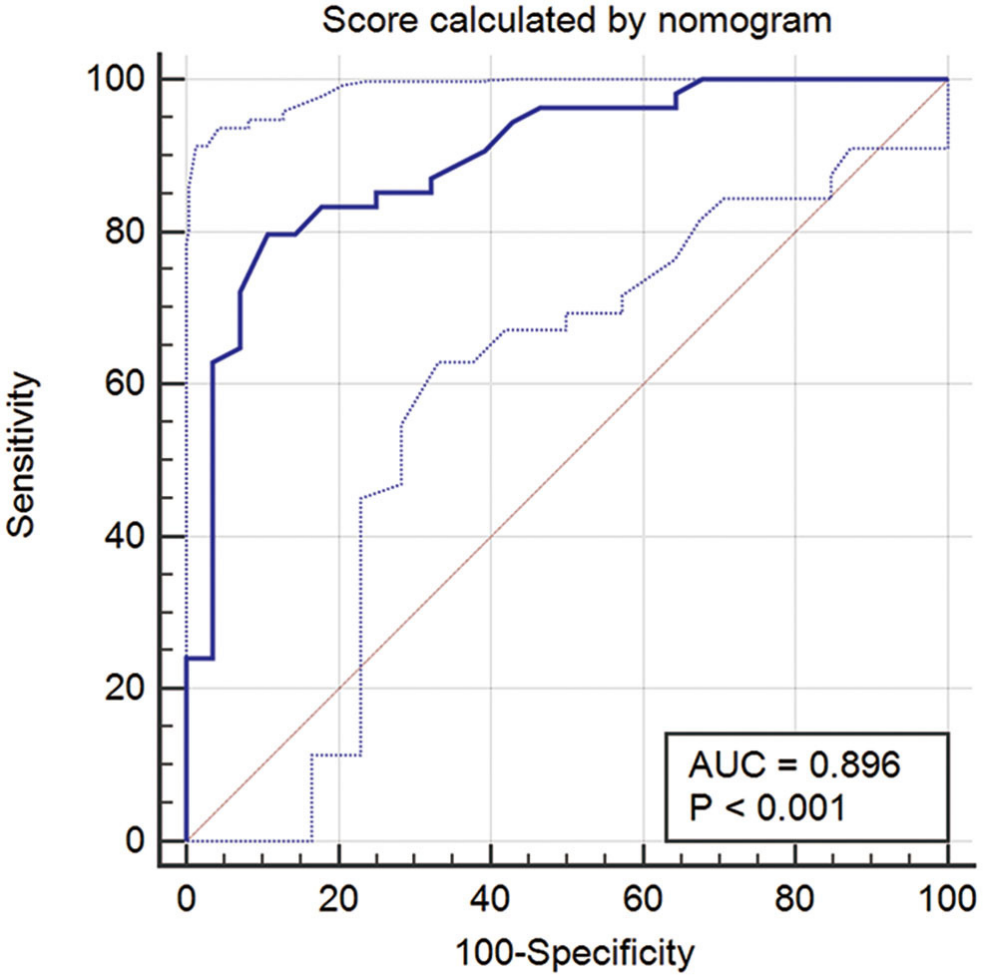
ROC curve when using the score calculated by the nomogram to predict NSCLC. ROC, receiver operator characteristic; NSCLC, non-small cell lung cancer.

For further investigation and clinical use, a nomogram was constructed that incorporated the miRNA panel and seven other risk factors to predict malignant disease (Figure 5A). A total score was calculated with the use of the miRNA panel, sex, age, smoking index, CT diagnosis, CEA test result, pleural tag, and solid proportion of lesions. The score of each factor is shown on the point calibration axis. The total points were calculated by adding the scores of each factor to estimate the possibility of malignant disease. The performance of the nomogram was also evaluated. A calibration curve of the nomogram is shown in Figure 5B, and it demonstrates that the NSCLC probability predicted by the nomogram accorded well with the actual probability. When the score calculated by the nomogram was used to distinguish NSCLC from benign disease, the AUC was 0.896 (*p* < 0.001), which was higher than that when using any other diagnostic method alone (Figure 6).

## Discussion

The prognosis of NSCLC patients depends mostly on the pathological stage, with a sharp decline from 68 to 92% for stage I/II patients to 1 to 13% for advanced-stage patients. However, only 16% of lung cancer patients are diagnosed at an early stage owing to the lack of effective methods (17). Therefore, it is urgent to discover accurate non-invasive biomarkers for early diagnosis to improve overall survival.

Conventionally, LDCT is used for the early detection of lung cancer. However, there are some arguments regarding the efficacy of LDCT in lung cancer screening owing to the high false-positive rate of LDCT in detecting small nodules. LDCT may lead to overdiagnosis and overtreatment, which would not increase the patient's life expectancy (18). In addition, changes in the levels of several serum-based protein biomarkers, such as CA-125, Cyfra21-1, CEA, and NSE, are used for early diagnosis. However, their sensitivity and specificity are limited, with sensitivities of 33.3, 11.1, 11.1, and 0% for CA-19-9, Cyfra21-1, CA-125, and CEA, respectively, for the early detection of stage I NSCLC (8). Some studies indicated that the amount of cfDNA is lower in patients with benign disease than those with cancer (19). However, the amount of cfDNA was demonstrated to be associated with tumor burden, which limits the application of cfDNA in early detection of lung cancer (20). With the similar genomic characteristics to the corresponding tumor, cfDNA is often used to detect genetic mutation for patients whose tissue analysis is inadequate on tissue specimens (21). The circulating miRNA levels are crucial diagnostic indicators owing to their non-invasive nature. Additionally, miRNAs present several characteristics that result in effective diagnostic measures. For example, it would be more inexpensive and convenient to test the levels of miRNAs than the current screening methods. Furthermore, circulating miRNAs were proven to be highly stable, and their tissue- or disease-specific properties helped improve the diagnostic accuracy when identifying malignant lesions from benign nodules that were shown by CT in a high-risk population (22, 23). Therefore, we further aimed to develop a nomogram based on the miRNA panel that could identify patients with early-stage NSCLC when detecting small nodules by a CT examination. The nomogram may be a useful tool for the preoperative prediction of lung nodules, improving the accuracy of lung cancer diagnosis and helping patients obtain prompt medical treatment.

Initially, candidate miRNAs were selected from more than 100 cancer-related miRNAs, and we found that a 12-miRNA panel (miRNA-17, miRNA-146a, miRNA-200b, miRNA-182, miRNA-155, miRNA-221, miRNA-205, miRNA-126, miRNA-7, miRNA-21, miRNA-145, and miRNA-210) could efficiently discriminate malignant lesions from small lung nodules (24–26). For convenience in the clinic, we formulated a cutoff value to distinguish lung cancer from benign disease and transformed the expression level of the miRNAs to dichotomous data. Our test demonstrated that under the current cutoff value, our miRNA panel presented higher sensitivity and specificity and a higher PPV than commonly used biomarkers. More importantly, the current study showed that the miRNA panel had significantly higher specificity and a significantly higher PPV than CT in clarifying lung lesions. Furthermore, to combine the advantages of different diagnostic methods, we built a nomogram that combines the panel and radiological features to improve the prediction accuracy.

Our study found that the expression of miRNA-17 was significantly upregulated in lung cancer patients compared with healthy controls, suggesting that miRNA-17 might have considerable diagnostic value in NSCLC. Similarly, a previous study reported that the level of exosomal miRNA-17-5p was upregulated in NSCLC samples, and it may be a novel non-invasive marker in the diagnosis of lung cancer (27). miRNA-182, miRNA-200b, and miRNA-205 have been reported as promising biomarkers for the early detection of NSCLC (28). In our study, miRNA-182, miRNA-200b, and miRNA-205 exhibited concordant increased plasma expression in cancer patients, indicating that these miRNAs could serve as useful biomarkers for the early diagnosis of NSCLC.

A previous study reported that the serum levels of miRNA-221 and miRNA-146b were reduced in NSCLC patients (29). In contrast, the current study demonstrated that the expression of miRNA-221 and miRNA-146a was significantly increased in the plasma of NSCLC patients. We deduced that the expression levels in the plasma do not correlate properly with those in the serum.

MiRNA-145 and miRNA-126 have been identified as tumor suppressor miRNAs that negatively regulate the proliferation and migration of cells and inhibit the progression and metastasis of cancers in lung tissue (30, 31). Paradoxically, our tests yielded contradictory results. The expression of miRNA-145 and miRNA-126 in plasma was maintained at higher levels in lung cancer patients than in healthy controls, indicating the potential protective effects of these two miRNAs in lung carcinoma. Identical to our study, Wang et al. reported that serum miRNA-145 was upregulated in NSCLC patients compared with healthy controls (32). Furthermore, Barshack et al. reported that miRNA-126 showed high expression levels in cancers metastasizing to the lung (33). We hypothesize that miRNA-145 and miRNA-126 may have various functions in different tumor stages and play different roles in tissue and in the circulation, and we also hypothesize that the different results in different studies may be related to the limited sample sizes and different study populations. Our study included only stage I lung cancer patients, and most of the tumors were <2 cm in size.

Previous studies have demonstrated the multiple functions of miRNA-7 in different tumor types, acting as both a promoter and a suppressor in cancers. Several studies have reported that miRNA-7 plays a suppressive role in human cancers, such as hepatocellular carcinoma, cervical cancer, breast cancer, and colorectal cancer. Instead, some studies have found that miRNA-7 is involved in the oncogenic effect of renal cell carcinoma and ovarian cancer (30, 34, 35). However, few studies have demonstrated the expression level of miRNA-7 in lung cancer. In our study, miRNA-7 was significantly upregulated in lung cancer patients, suggesting that miRNA-7 may act as an oncogene in lung cancer.

MiRNA-210 is involved in different pathways, such as cell cycle regulation, cell proliferation, apoptosis, metabolism, and metastasis (30, 36). Therefore, any alteration or modification in the structure and expression of miRNA-210 could result in abnormal functions. The current study reported that the level of miRNA-210 was increased in cancer samples, and it could serve as a potential non-invasive biomarker for the early diagnosis of lung cancer. The majority of studies have found that miRNA-210 exhibits oncogenic properties and is upregulated in several cancers, such as breast cancer, pancreatic cancer, and glioblastoma (36, 37).

Previous studies have presented miRNA-based prediction nomograms for predicting lymph node metastases in breast cancer (38). To our knowledge, however, there has been no such study for predicting benign and malignant disease in small lung nodules. The current study was the first to investigate the accuracy of a 12-miRNA panel as an effective, non-invasive method for the preoperative evaluation of small nodules in the lung. Our study showed that the sensitivity of the miRNA panel was higher than that of the common tumor markers. A nomogram is a visual statistical model that is developed to optimize the predictive accuracy of individuals. Preoperative nomograms can help clinicians specifically diagnose small tumors. In the current study, considering the high sensitivity of CT in detecting lung lesions and the high specificity and PPV of the miRNA panel, we built a non-invasive nomogram model incorporating the plasma miRNA panel and clinical and imaging features that might improve the efficacy of lung nodule diagnosis, and the AUC was 0.896. The calibration plots presented acceptable agreement between the predicted and actual probabilities in the validation cohort, guaranteeing the reliability and repeatability of the nomogram.

The strengths of the current study were that it investigated specifically stage I NSCLC patients and discussed the diagnostic accuracy of combining the miRNA signature and imaging features. Considering the non-invasive characteristics of miRNA markers for stage I patients, the plasma-based miRNA panel could be applied to clinical practice and help reduce the misdiagnosis and overtreatment of low-risk disease. In addition, the miRNA panel may help identify high-risk individuals who should undergo further examinations, including LDCT. To the best of our knowledge, this was the first study to construct a nomogram combining a miRNA panel and imaging characteristics for the prediction of lung cancer. However, we are also aware of some limitations to this study that need to be noted. First, the sample size was not very large. Second, although internal validation was performed to validate the predictive accuracy of our nomogram, it is better to have external validation in different medical centers. Thus, further large-scale multicenter prospective studies are still needed to be conducted to explore the relationship between miRNA panel and different histological types and further validate the potential diagnostic value of our miRNA panel. The predictive value of our miRNA panel in genetic mutation state and survival outcomes should also be studied in the future.

## Conclusions

In summary, we presented a 12-miRNA panel as a non-invasive plasma biomarker that can effectively improve the accuracy of the identification of small lung nodules. Additionally, we constructed a predictive nomogram based on the miRNA panel and imaging characteristics for the early diagnosis of lung cancer. Our miRNA panel and predictive model exhibit excellent potential for the diagnosis of early-stage NSCLC and could be of use to clinicians.

## Data Availability Statement

The datasets generated for this study are available on request to the corresponding author.

## Ethics Statement

This retrospective study was reviewed and approved by the Institute Research Medical Ethics Committee of Sun Yat-sen University Cancer Center, the reference number is B2018-011. The patients/participants provided their written informed consent to participate in this study.

## Author Contributions

Conception and design were carried out by WW, DC, XZ, LL, and LZ. Administrative support was given by WW, LL, and LZ. WC and ZX provided the study materials and recruited the patients. ZH, WC, KX, and RZ collected and assembled the data. WW, GW, and DZ analyzed and interpreted the data. All authors wrote the manuscript. The final approval of the manuscript was given by all authors.

## Conflict of Interest

The authors declare that the research was conducted in the absence of any commercial or financial relationships that could be construed as a potential conflict of interest.

## Supplementary Material

Figure S1 ROC curve of each miRNA in the training group. **(A-L)**. ROC curves of miRNA-17, miRNA-146a, miRNA-200b, miRNA-182, miRNA-155, miRNA-221, miRNA-205, miRNA-126, miRNA-7, miRNA-21, miRNA-145, and miRNA-210 in the training group. *AUC*, area under the curve; *ROC*, receiver operator characteristic.

Figure S2 ROC curve when combining the 12 miRNAs after being transformed to dichotomous data. *AUC*, area under the curve; *ROC*, receiver operator characteristic.

Table S1 Sensitivity, specificity, and positive predictive value of the 12-miRNA panel compared with those of traditional tumor markers.

Table S2 Sensitivity, specificity, and positive predictive value of the 12-miRNA panel compared with those of CT diagnosis.

## Abbreviations

AUC: area under the curve
CEA: carcinoembryonic antigen
CT: computed tomography
GGO: ground-glass opacity
LDCT: low-dose computed tomography
NSCLC: non-small cell lung cancer
NSE: neuron-specific enolase
PCR: polymerase chain reaction
PPV: positive predictive value
RT: reverse transcription
ROC: receiver operating characteristic
SCLC: small cell lung cancer
SD: standard deviation.
