# Supplementary figures and images for: Early Detection of Non-Small Cell Lung Cancer by Using a 12-microRNA Panel and a Nomogram for Assistant Diagnosis
Source: Front Oncol. 2020 Jun 11;10:855. doi: 10.3389/fonc.2020.00855 (PMC7301755; doi:10.3389/fonc.2020.00855)

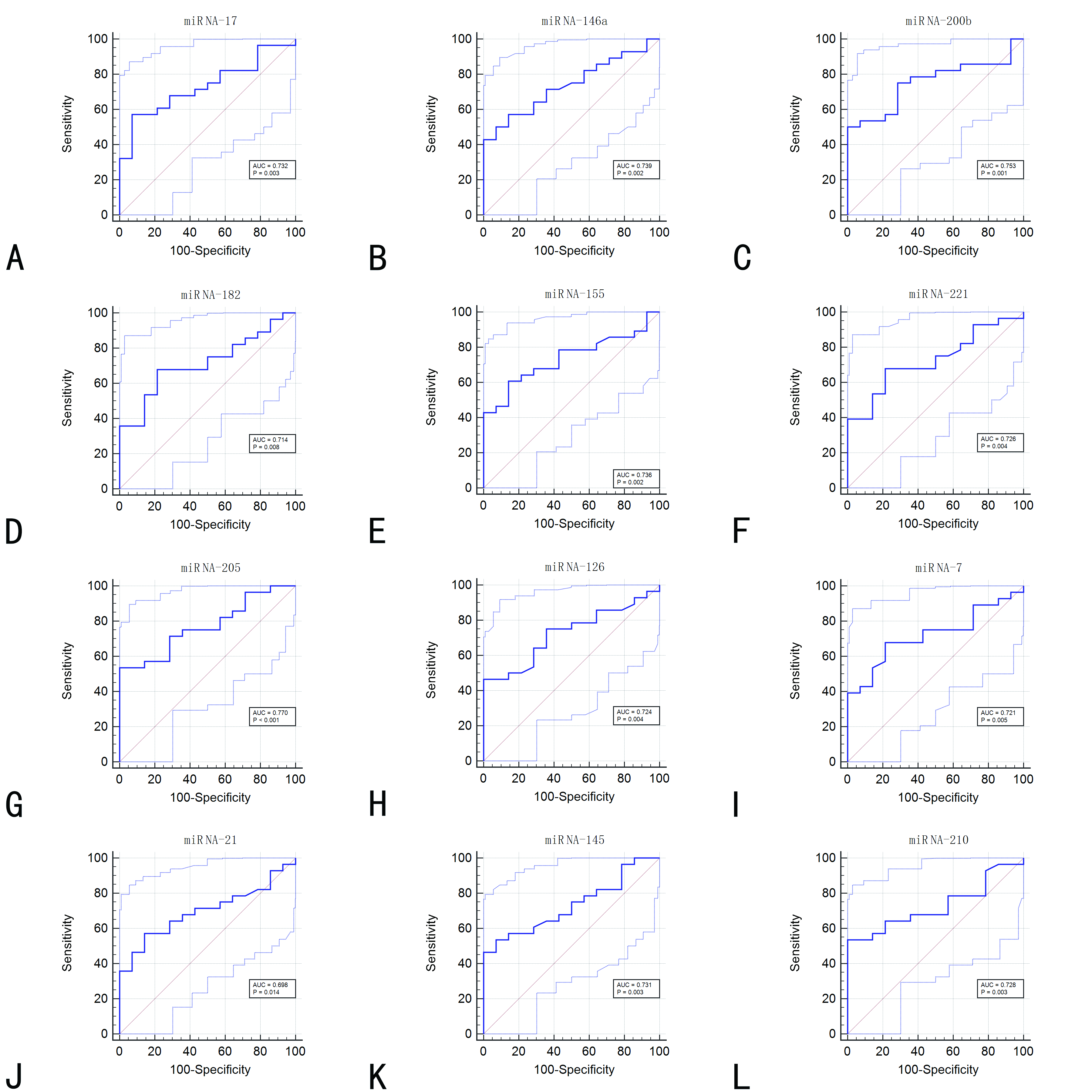

Supplement: Figure S1 — ROC curve of each miRNA in the training group. (A-L). ROC curves of miRNA-17, miRNA-146a, miRNA-200b, miRNA-182, miRNA-155, miRNA-221, miRNA-205, miRNA-126, miRNA-7, miRNA-21, miRNA-145, and miRNA-210 in the training group. AUC, area under the curve; ROC, receiver operator characteristic. [file Image_1.TIF]

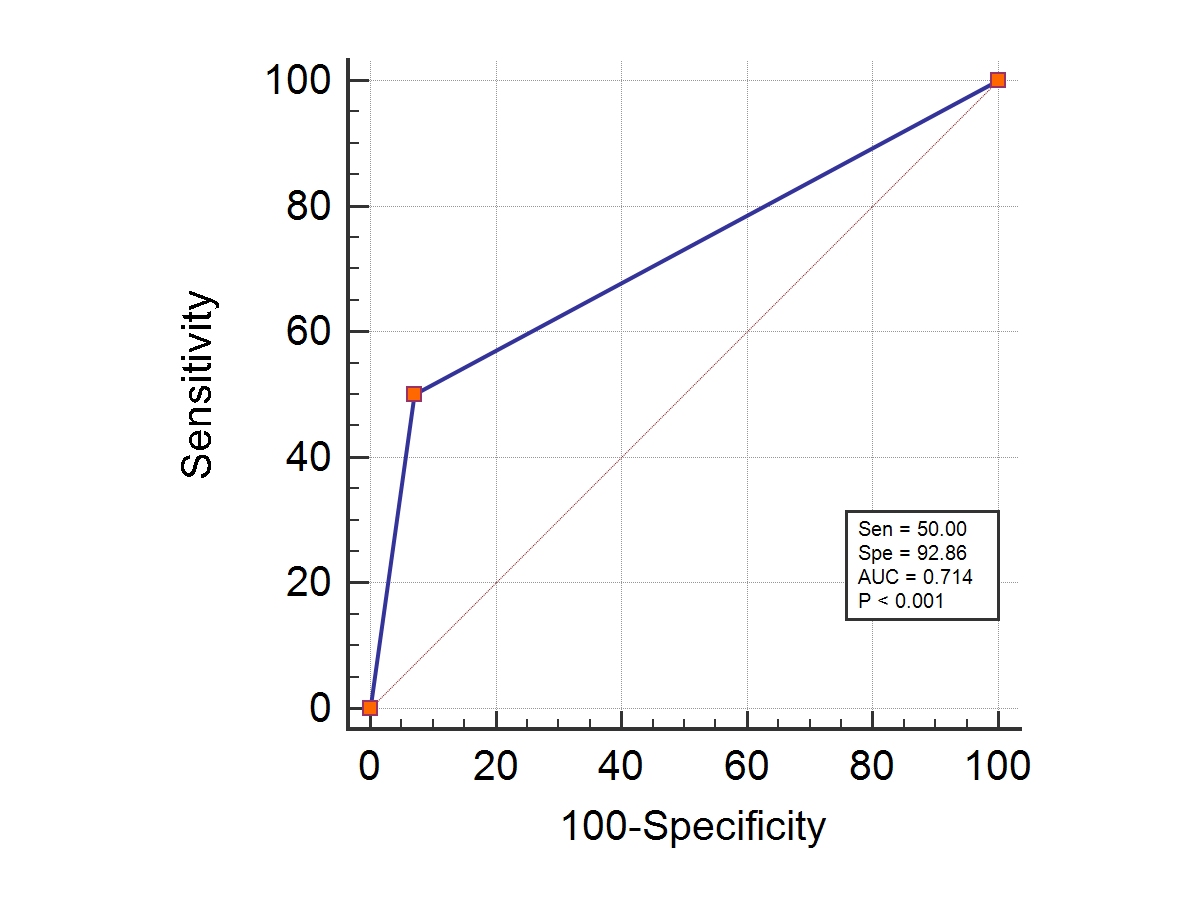

Supplement: Figure S2 — ROC curve when combining the 12 miRNAs after being transformed to dichotomous data. AUC, area under the curve; ROC, receiver operator characteristic. [file Image_2.TIF]
