# Supplementary material for: Early Detection of Non-Small Cell Lung Cancer by Using a 12-microRNA Panel and a Nomogram for Assistant Diagnosis
Source: Front Oncol. 2020 Jun 11;10:855. doi: 10.3389/fonc.2020.00855 (PMC7301755; doi:10.3389/fonc.2020.00855)
Supplement: Table S1 — Sensitivity, specificity, and positive predictive value of the 12-miRNA panel compared with those of traditional tumor markers. [file Table_1.DOCX]

**Supplementary** **Table 1. Sensitivity, specificity and positive predictive value of the 12-miRNA panel compared with those of traditional tumor markers.**

| **Diagnostic method** | **Sensitivity** | ***P value*** | **Specificity** | ***P value*** | **PPV** | ***P value*** |
| --- | --- | --- | --- | --- | --- | --- |
| **12-miRNAs panel** | 42.6 | *Reference* | 96.4 | *Reference* | 95.8 | *Reference* |
| **CEA** | 18.5 | *0.011* | 92.9 | *1.000* | 83.3 | *0.253* |
| **Cyfra21-1** | 33.3 | *0.424* | 67.9 | *0.021* | 66.7 | *0.012* |
| **NSE** | 0.0 | *<0.001* | 96.4 | *1.000* | 0.0 | *<0.001* |
| *CEA*, carcinoembryonic antigen; *NSE*, neuron specific enolase; *PPV*, positive predictive value. | | | | | | |
