# Supplementary material for: Early Detection of Non-Small Cell Lung Cancer by Using a 12-microRNA Panel and a Nomogram for Assistant Diagnosis
Source: Front Oncol. 2020 Jun 11;10:855. doi: 10.3389/fonc.2020.00855 (PMC7301755; doi:10.3389/fonc.2020.00855)
Supplement: Table S2 — Sensitivity, specificity, and positive predictive value of the 12-miRNA panel compared with those of CT diagnosis. [file Table_2.DOCX]

**Supplementary Table 2. Sensitivity, specificity and positive predictive value of the 12-miRNA panel compared with those of CT diagnosis.**

| **Characteristic** | **Sensitivity （%）** | | | **Specificity（%）** | | | **PPV（%）** | | |
| --- | --- | --- | --- | --- | --- | --- | --- | --- | --- |
|  | **miRNA** | **CT** | ***P Value*** | **miRNA** | **CT** | ***P Value*** | **miRNA** | **CT** | ***P Value*** |
| **Full cohort** | 42.6 | 74.1 | *0.020* | 96.4 | 53.6 | *<0.001* | 95.8 | 75.5 | *<0.001* |
| **Composition** |  |  |  |  |  |  |  |  |  |
| Pure GGO | 42.9 | 61.9 | *0.424* | 88.9 | 55.6 | *0.250* | 90.0 | 76.5 | *0.621* |
| Mix GGO/ solid | 42.4 | 81.8 | *0.001* | 100.0 | 52.6 | *<0.001* | 100.0 | 75.0 | *0.010* |
| **Diameter** |  |  |  |  |  |  |  |  |  |
| ≤2cm | 37.2 | 69.8 | *0.007* | 95.2 | 47.6 | *0.002* | 94.1 | 73.2 | *0.050* |
| >2cm | 63.6 | 90.9 | *0.375* | 100.0 | 71.4 | *<0.001* | 100.0 | 83.3 | *0.509* |
| *CM*, centimeter; *CT*, computed tomography; *GGO*, ground glass opacity; *PPV*, positive predictive value. | | | | | | | | | |
